# A New Technique in Primary Repair of Congenital Esophageal Atresia Preventing Anastomotic Stricture Formation and Describing the Opening Condition of Blind Pouch: Plus (“+”) Incision

**DOI:** 10.1155/2011/527323

**Published:** 2011-05-17

**Authors:** Mehmet Melek, Ufuk Cobanoglu

**Affiliations:** ^1^Department of Pediatric Surgery, University of Yuzuncu Yil Medical Faculty, 65300 Van, Turkey; ^2^Department of Thoracic Surgery, University of Yuzuncu Yil Medical Faculty, 65300 Van, Turkey

## Abstract

Anastomotic strictures are common and important problems following repair procedures of esophageal atresia. We hereby defined an anastomosis technique that could efficiently prevent this complication in 11 patients with esophageal atresia (EA) and tracheoesophageal fistula (TEF). The proximal end of the atretic esophagus was opened with a plus (“+”)-shaped incision providing sufficient anastomosis width. Longitudinal incisions of 2 mm length were made on the anterior and posterior parts of the distal end according to the patients. The two ends were anastomosed with a primary suture at a single plain. We performed this technique on 11 patients, and in the 4-year follow-up period no dilatation proved necessary in any of our patients due to anastomotic strictures or symptomatic dysphagia. This technique that we have described provides a large zigzag anastomosis line and in this way minimizes the incidence of stricture formation. Furthermore, this technique, which we believe to have provided a new opinion on the topic of how to open the proximal end of an atretic esophagus, is quite easy and effective.

## 1. Introduction

The incidence of anastomotic strictures following the repair of esophageal atresia (EA) is very high, reaching 35–55% in some series [1–5]. A circular anastomosis line compressed onto one plan is the most important factor increasing the probability of development of this complication [6]. There is insufficient data in the literature about how the atretic esophageal pouch should be opened. The technique we will describe does not increase the distance between the ends of the pouch and does not lead to anastomotic tenseness, since it does not result in tissue loss in the blind pouch ends. Moreover, it minimizes stricture development since it provides a large anastomosis line which is not in one plain.

## 2. Material and Method

Eleven cases operated for the diagnosis of esophageal atresia and tracheoesophageal fistulae between the years of 2005–2009 were evaluated. These 11 cases with proximal EA and accompanying distal tracheaesophageal fistulae (TEF) had undergone the operative procedure utilizing the described technique by the same surgeon. Six of the cases had had low birth weights with a mean birth weight of 2453.63 ± 575.45 grams. The minimum gestational age was 32 weeks. In the evaluation of cases according to the Waterston risk grouping, 4 cases were in the A group, 2 were in B1, 3 were in B2, and 2 were in the C2 group. In 1962, Waterston developed a prognostic classification system for esophageal atresia that is still used today. Category A includes patients who weigh more than 5.5 lb (2.5 kg) at birth and who are otherwise well; category B includes patients who weigh 4–5.5 lb (1.8–2.5 kg) and are well or who have higher birth weights, moderate pneumonia, and congenital anomalies; category C includes patients who weigh less than 4 lb (1.8 kg) or have higher birth weights, severe pneumonia, and severe congenital anomalies [7].

The variants of esophageal atresia have been described using many anatomic classification systems. To avoid ambiguity, the clinician should use a narrative description. Nevertheless, Gross of Boston described the classification system that is most often cited. According to this system, the types of esophageal atresia and the approximate incidence in all infants born with esophageal anomalies are as follows: Type A—Esophageal atresia without fistula or the so-called pure esophageal atresia (10%), Type B—Esophageal atresia with proximal TEF (<1%), Type C—Esophageal atresia with distal TEF (85%), Type D—Esophageal atresia with proximal and distal TEFs (<1%), Type E—TEF without esophageal atresia or the so-called H-type fistula (4%), and Type F—Congenital esophageal stenosis (<1%) [8]. All of our cases were in group Type C according to the Gross classification. The gap lengths between the proximal and distal ends ranged from 0.5 cm to 3 cm (Table 1).

**Figure 1:**
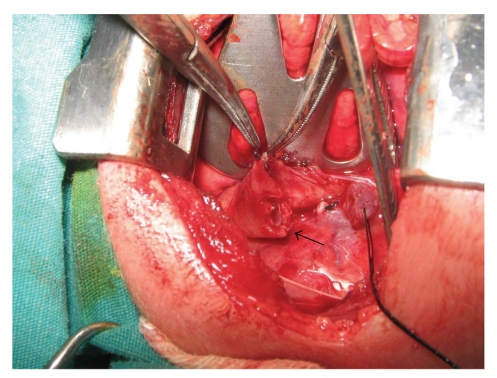
The proximal esophageal pouch opened with plus “+”—shaped incision.

**Figure 2:**
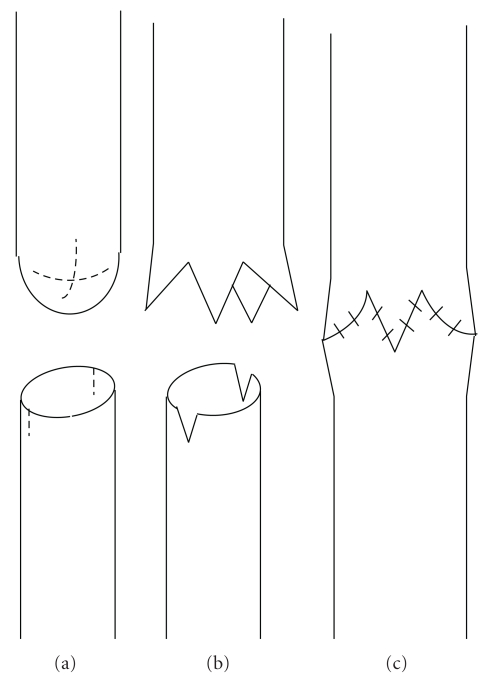
(a) Plus incision onto the blind pouch and small incisions to the distal esophagus bilaterally, (b) the opened state of both ends after incision, (c) view of the anastomosed ends.

The operation was performed through the classical right thoracotomy technique extrapleurally. First, TEF was tied and the lower pouch was freed. After complete mobilization of the proximal end, the upper pouch was opened with a plus “+”-shaped incision (Figure 1). Longitudinal incisions of 2 mm length, oblique to the transverse section of the esophagus, were made on the anterior and posterior parts of the distal end with the patient in supine position. The ends were then brought together, and all layers (including the esophageal mucosa) were primarily single-point sutured with 5/0 monofilament polyglyconate synthetic absorbable suture (Manufacturer: US Surgical) in single file (Figures 2(a), 2(b), and 2(c)). 

Esophagus passage radiographies were performed in all cases at the first month after operation. The patients were evaluated in follow-up examinations at regular intervals.

## 3. Results

The postoperative hospital stay was 9.54 ± 3.14 days. Feeding by mouth was possible in 6.36 ± 2.73 days (min 3 days, max 12 days) on average (Table 1). Early complications of esophageal atresia surgery such as anastomotic leak, recurrent tracheoesophageal fistula, or anastomotic stricture did not occur in any of our patients.

In one patient with gastroesophageal reflux (GER) disease who did not respond to medical treatment, antireflux surgery using the Nissen fundoplication technique was performed at the 12th month. In addition, other late complications such as esophageal dysmotility and tracheomalacia were not observed in any of the cases. 

**Figure 3:**
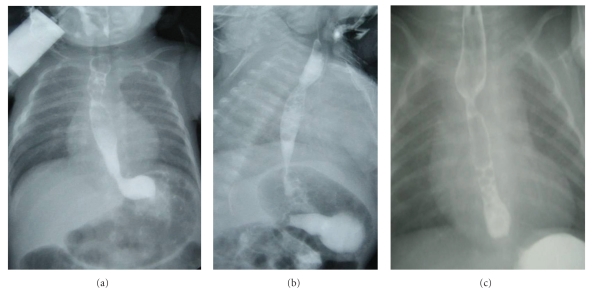
(a) Normal esophageal passage X-ray view of the case with no anastomotic stricture. (b) Nonsymptomatic moderate narrowing and (c) serious anastomotic stricture that caused expansion of the proximal esophagus (another case of our patients).

We did not observe dysphagia in any of our patients in a mean follow-up period of 2, 41 ± 0, and 58 years (min 1.5 years, max 3 years). Postoperative esophageal passage images were normal supporting the patients normal clinical condition during followup (Figure 3(a)). Although there was a moderate narrowing on the radiographic evaluation of 1 patient (Figure 3(b)), the patient did not have any symptoms, and in the followup for a long period, he did not experience dysphagia even for solid foods. There was not a view of serious anastomotic stricture in any case (Figure 3(c)).

## 4. Discussion

Anastomotic strictures are still the most common complications of the anastomosis area of esophageal atresia repair [1–6, 9, 10]. The suture material, type of anastomosis, anastomotic tension, ischemia, anastomotic leak, and the presence of GER are the main factors affecting the development of anastomotic strictures [6, 11]. 

The classical surgical repair technique for esophageal atresia is end to end anastomosis. Since anastomotic stricture is an important problem in esophageal atresia repair operations, many techniques have been defined to prevent it. The main aims of these techniques are to obtain an anastomosis line which is nontense, large and unrestricted to one plain [12, 13]. The end-to-side anastomosis technique described by Sulamaa et al. is one of the first anastomosis techniques in preventing this complication [14]. The technique described by Singh and Shun also depends on obtaining a large, unrestricted to a single-plain anastomosis line [6]. Although end-to-side anastomoses produce a large anastomosis line, since this anastomosis line is restricted to one plain, they may undermine the formation of anastomotic strictures. Due to this reason, anastomosis techniques providing a large and unrestricted to one plain anastomosis line minimize the risk of narrowing in the healing period [6]. 

This technique that we have described at the lower and upper ends of atretic esophagus seems to be an alternative way of opening the blind pouch in esophageal atresia repair. With this technique, 4 separate flap-like extensions are formed at the end of the plus-shaped opened esophagus (Figure 2(b)), and since these extensions can relax more easily than the flat-line opened esophageal ends, the space loss between the pouches can be minimized. We think that with the technique that we used while opening the proximal end, relative prevention of tissue loss may provide length gain. Furthermore, two flaps are located at the proximal end to the formed notches with the bilateral incisions in the anterior, posterior, and oblique plains; the other free edges of the lower end are joined to the lateral flaps which dilate the lumen by stretching the distal esophagus, which are relatively narrower than the upper pouch lumen, bilaterally. The joining of the edges of the distal esophageal end in the patient's sagittal plain with the flaps corresponding to them without any incisions renders the lumen to remain large, with continuation of the zigzag suture line in a curved suture line (Figure 2(c)). Compatible with the main aim of the technique described by Singh and Shun providing a circular anastomosis line in the oblique plain, our technique also renders possible to obtain a zigzag anastomosis line that is not restricted to one line. 

In conclusion, we described a technique which is an easy and suitable way of opening the blind esophageal pouch. This blind pouch opening technique contributes to the shortening of the distance between the esophageal ends. Although the reported serial contains a limited number of patients, it has been thought that providing a large and “unrestricted to one plane anastomosis” line, it minimizes the formation of stricture. It seems to be a promising, easy technique in preventing stricture formation which can be used in primary anastomosis of newborns with esophageal atresia and tracheoesophageal fistulae that have short distances between the two esophageal ends. With the increased number of patients and prolonged follow-up periods, a more accurate decision on this topic may be made.

## Figures and Tables

**Table 1:** Characteristics of cases.

Case number	Sex	Gestational age (Week)	Birth weight (gr)	Anatomical Classification (Gross)	Risk group (Waterston's)	Gap distance (cm)	Following time (year)
1	Female	38	2210	Type C	B_2_	1.5	2.5
2	Female	40	3500	Type C	A	1	1.5
3	Male	39	3100	Type C	B_2_	2.5	2
4	Male	38	2810	Type C	A	0.5	1.5
5	Female	37	1800	Type C	B_2_	2	3
6	Female	40	2320	Type C	B_1_	0.5	3
7	Female	38	2700	Type C	A	1.5	2
8	Female	40	1800	Type C	B_1_	2	2.5
9	Female	37	2750	Type C	C_2_	3	3
10	Female	36	1700	Type C	A	1.5	3
11	Male	32	2300	Type C	C_2_	2.5	2.5
